# The transcription factor NRF1 (NFE2L1) activates aggrephagy by inducing p62 and GABARAPL1 after proteasome inhibition to maintain proteostasis

**DOI:** 10.1038/s41598-023-41492-9

**Published:** 2023-09-01

**Authors:** Atsushi Hatanaka, Sota Nakada, Gen Matsumoto, Katsuya Satoh, Iori Aketa, Akira Watanabe, Tomoaki Hirakawa, Tadayuki Tsujita, Tsuyoshi Waku, Akira Kobayashi

**Affiliations:** 1 Laboratory for Genetic Code, Graduate School of Life and Medical Sciences, Doshisha University, 1-3 Tatara Miyakodani, Kyotanabe, Kyoto 610-0394 Japan; 2 Research Fellow of Japan Society for the Promotion of Science, Tokyo, Japan; 3 Laboratory for Genetic Code, Department of Life and Medical Sciences, Doshisha University, Kyotanabe, Kyoto Japan; 4 Department of Anatomy and Neurobiology, Nagasaki University School of Medicine, Nagasaki, Japan; 5 Graduate School of Medicine, Kyoto University, Kyoto, Japan; 6 Laboratory of Biochemistry, Faculty of Agriculture, Saga University, Saga, Japan; 7 The United Graduate School of Agricultural Sciences, Kagoshima University, Kagoshima, Japan

**Keywords:** Biochemistry, Molecular biology

## Abstract

The ubiquitin‒proteasome system (UPS) and autophagy are the two primary cellular pathways of misfolded or damaged protein degradation that maintain cellular proteostasis. When the proteasome is dysfunctional, cells compensate for impaired protein clearance by activating aggrephagy, a type of selective autophagy, to eliminate ubiquitinated protein aggregates; however, the molecular mechanisms by which impaired proteasome function activates aggrephagy remain poorly understood. Here, we demonstrate that activation of aggrephagy is transcriptionally induced by the transcription factor NRF1 (NFE2L1) in response to proteasome dysfunction. Although NRF1 has been previously shown to induce the expression of proteasome genes after proteasome inhibition (i.e., the proteasome bounce-back response), our genome-wide transcriptome analyses identified autophagy-related *p62*/*SQSTM1* and *GABARAPL1* as genes directly targeted by NRF1. Intriguingly, NRF1 was also found to be indispensable for the formation of p62-positive puncta and their colocalization with ULK1 and TBK1, which play roles in p62 activation via phosphorylation. Consistently, NRF1 knockdown substantially reduced the phosphorylation rate of Ser403 in p62. Finally, NRF1 selectively upregulated the expression of *GABARAPL1,* an ATG8 family gene, to induce the clearance of ubiquitinated proteins. Our findings highlight the discovery of an activation mechanism underlying NRF1-mediated aggrephagy through gene regulation when proteasome activity is impaired.

## Introduction

The ubiquitin‒proteasome (UPS) and autophagy are the two essential protein quality control systems that protect cells against the detrimental consequences of unfolded, misfolded, or damaged proteins that lead to severe imbalances in cellular functions and that maintain protein homeostasis (i.e., proteostasis). UPS and autophagy dysfunction is associated with aging and age-related human diseases, including neurodegeneration, cancer, and metabolic diseases, due to imbalanced proteostasis^1–3^. The UPS functions as the primary degradation system for small and short-lived proteins, which are conjugated with polyubiquitinated chains and degraded by the proteasome. The proteolytic capacity of this system can be diminished by a variety of external and internal stimuli, such as chemical inhibitors (e.g., bortezomib for multiple myeloma therapy), oxidative stress, aberrant or excessive protein contents, or aging. When proteasome functions are suppressed, cells maintain proteostasis through the activation of autophagy, through which large and potentially harmful protein aggregates are removed. In contrast, however, the inhibition of autophagy rarely activates the proteasome^1^. These findings provide strong evidence indicating functional communication between the UPS and autophagy in protein quality control; nevertheless, the molecular basis that governs the shift from the UPS to autophagy after proteasome inhibition remains to be fully elucidated.

Multiple lines of evidence demonstrate that the transcription factor NRF1 (NFE2L1) plays crucial roles in proteostasis. NRF1 belongs to the CNC transcription family, which also includes NRF2 (NFE2L2) and NRF3 (NFE2L3), and has been shown to regulate proteasome subunit gene expression after the proteasome becomes dysfunctional^4–6^. This cellular counter response is called the “proteasome bounce-back response” (or “proteasome recovery”). Under physiological conditions, the transcriptional activity of NRF1 is suppressed by its sequestration in the endoplasmic reticulum (ER) and the E3 Ub ligase HRD1-mediated proteasomal degradation in the endoplasmic reticulum-associated protein degradation (ERAD) system^7–9^. After proteasome suppression, NRF1 undergoes cleavage by the DDI2 enzyme, resulting in NRF1 release from the ER and subsequent nuclear translocation^10, 11^. Then, NRF1 facilitates the restoration of proteasome activity by upregulating proteasome gene expression by its binding with small Maf proteins to an antioxidant response element (ARE) . Supporting these findings, neuron-specific *Nrf1* deletion led to mice exhibiting neurodegeneration disease accompanied by the accumulation of ubiquitinated proteins (hereafter referred to as Ub-proteins) due to the reduced proteasome activity^12, 13^. Importantly, the proteasome bounce-back response exhibits significant medical relevance because therapeutic proteasome inhibition by bortezomib promptly triggers the upregulation of proteasome genes in cancer cells, such as multiple myeloma cells, leading to cancer cell resistance to anticancer drugs^14–19^. Nevertheless, whether NRF1 enhances other protein degradation systems, such as autophagy, when proteasome activity is fully suppressed is unclear.

Autophagy was initially identified as a nonselective and bulk degradation system of cellular compartments, including protein aggregates or organelles, under nutrient or growth factor deprivation^1, 3^. The cytoplasmic substrates are engulfed by the phagophore, which closes to form an autophagosome, and after fusing with lysosomes, the autophagosome and contents are degraded. However, recent research advances have highlighted that cytoplasmic substrates can be selectively degraded by autophagy, and this degradation pathway is called “selective autophagy”, which is augmented by several stress signals^1^. For example, proteasome inhibition promotes “aggrephagy”, a type of selective autophagy that eradicates protein aggregates^20^. The selectivity of aggrephagy is governed by the binding affinity of autophagy receptors for substrates^3, 21^. Among these receptors, the p62 protein, also called sequestosome 1 (SQSTM1), plays two critical roles in aggrephagy, that is, the formation of p62-positive puncta (i.e., sequestosomes) and their subsequent sequestration by phagophores. First, p62-positive puncta are formed in multiple sequential steps. Through the ubiquitin-associated (UBA) domain, p62 associates with accumulated Ub-proteins, stimulating the ULK1-mediated phosphorylation of p62 at Ser409, also in the UBA domain^22^. This phosphorylation modification facilitates the transition of p62 from a dimer that forms through the UBA domain to a monomer and the phosphorylation at Ser403 by ULK1, TBK1 or CK2^22–25^. Thus, p62 shows enhanced binding affinity for Ub-proteins, and their binding results in the phase separation formation of sequestosomes^26–28^. Second, these sequestosomes are sequestrated by the phagophore through its direct interaction with ATG8 proteins and are subsequently subjected to autophagy-related proteolysis. These findings strongly suggest essential roles for p62 in aggrephagy and that ubiquitin is required for not only effective proteolysis via the UPS but also for aggrephagy.

The ATG8 family proteins are key molecules in the formation of autophagosomes because they conjugate with the lipid phosphatidylethanolamine (PE) and thus associate with autophagosomal membranes^3, 29, 30^. In mammals, the ATG8 family consists of seven orthologs (LC3A, LC3B, LC3B2, LC3C, GABARAP, GABARAPL1 and GABARAPL2) that are classified into two subfamilies defined on the basis of their amino acid sequence similarities: the LC3 and GABARAP subfamilies. Their functional similarities and differences have been reported, although further investigation is required to fully confirm these reports^21, 30^. GABARAPL1 and other ATG8 family proteins directly associate with the autophagy receptors p62, NBR1 autophagy cargo receptor 1 (NBR1) and Optineurin (OPN), recruiting sequestosomes to forming autophagic vesicles. Moreover, GABARAPL1 has also been described to be involved in activating autophagosome formation by directly interacting with ATG2 and ULK1^31, 32^. Nevertheless, further extensive investigation is necessary to fully characterize the functional roles played by GABARAPL1 in aggrephagy.

Thus, we sought to comprehensively investigate the molecular mechanisms by which impaired proteasome function activates aggrephagy. To achieve this goal, we focused on the transcription factor NRF1, a major regulator of the proteasome bounce-back response, and discovered that NRF1 was involved in regulating this transitional mechanism. NRF1 directly upregulated the expression of the autophagy-related genes *p62* and *GABARAPL1,* which promoted the removal of Ub-proteins. Moreover, we found that NRF1 was crucial for the formation of p62-positive puncta. Thus, our present study highlights the crucial biological function of NRF1 in the activation of autophagy triggered by proteasome activity dysfunction.

## Results

### NRF1 promotes the clearance of Ub-proteins by activating autophagy after proteasome inhibition

**Figure 1:**
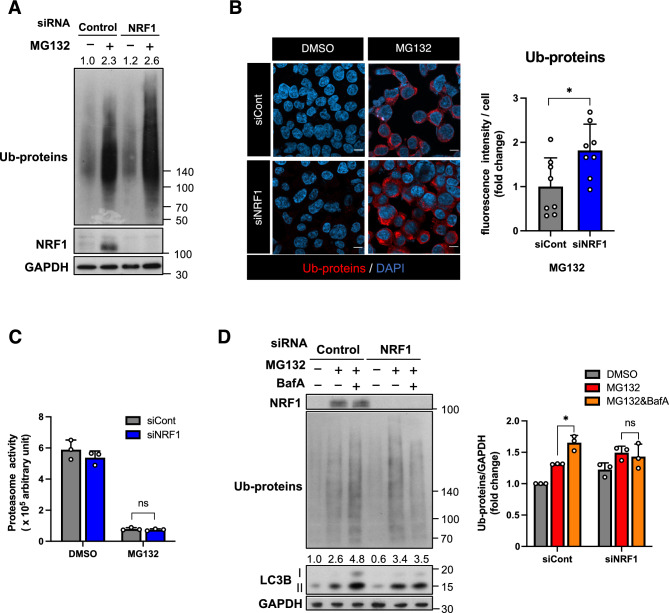
NRF1 activates lysosomal proteolysis of Ub-proteins in response to proteasome inhibition. (**A**) *NRF1* knockdown elicited the accumulation of ubiquitinated proteins (Ub-proteins) in HCT116 cells treated with the proteasome inhibitor MG132 (1 µM) for 16 h. A representative result from three-independent experiments is shown. The values represent the relative intensities of the bands of Ub-proteins normalized to the GAPDH level. (**B**) *NRF1* knockdown also increased fluorescence intensities of Ub-proteins in HCT116 cells treated with MG132 (1 µM) for 16 h. Ub-proteins were visualized in immunostaining using anti-ubiquitinated protein antibody (clone FK2). A right graph presents the quantification of fluorescence intensities of Ub-proteins using ImageJ in more than 150 cells (eight different views). Scale bar, 10 μm. (**C**) Treatment with MG132 (1 µM) for 16 h markedly repressed proteasome activity even in the presence of NRF1. The proteasome activity of HCT116 cells treated with the indicated reagents was measured by incubating 10 µg of whole-cell extracts with 2 mM ATP and the fluorogenic substrate Suc-LLVY-AMC (n = 3). (**D**) NRF1 promoted the lysosomal clearance of Ub-proteins under proteasome inhibition. The values represent the relative intensities of the bands of LC3-II proteins normalized to the GAPDH level. In (**A**–**D**), 2 days after siRNA transfection, HCT116 cells were treated with DMSO or MG132 (1 μM) in combination with DMSO or BafA (10 nM) for 16 h. (**A** and **D**) The levels of Ub-protein levels in immunoblots were quantified by ImageJ and normalized to the GAPDH level (n = 3). (**A**, **C** and **D**) ANOVA followed by Tukey’s test: mean ± SD, *p < 0.05, ns: not significant. (**B**) Welch t-test, *p < 0.05.

We initiated our investigation by exploring the possibility that NRF1 promotes autophagy-mediated protein degradation in response to proteasome inhibition. Human colorectal cancer HCT116 cells were transfected with *NRF1* siRNA following treatment with the proteasome inhibitor MG132. A subsequent immunoblot analysis revealed that MG132 treatment led to the accumulation of ubiquitinated proteins (Ub-proteins) in the cells (Fig. 1A). Further *NRF1* knockdown markedly increased the levels of accumulated Ub-proteins compared to the effect of MG132 treatment alone. Immunohistochemical staining showed that Ub-protein puncta were localized in the perinuclear region in the cells treated with MG132, and the fluorescence intensity of puncta was increased by *NRF1* knockdown (Fig. 1B). Under the experimental conditions of this proteasome inhibition, NRF1 failed to restore proteasome activity (Fig. 1C). These results allowed us to hypothesize that NRF1 activates alternate proteolytic systems, such as autophagy.

We next examined the biological relationship between NRF1 and autophagy after proteasome inhibition. HCT116 cells were treated with the autophagy inhibitor bafilomycin A1 (BafA) to repress lysosomal proteolysis, and then an immunoblot analysis was performed using an anti-ubiquitin antibody (Fig. 1D). Indeed, BafA treatment led to an increase in Ub-protein accumulation mediated by MG132, indicating that autophagy also contributed to the clearance of the Ub-proteins. Intriguingly, under these experimental conditions, further *NRF1* knockdown did not increase the Ub-protein level after BafA treatment (as shown in the right graph). These results strongly support our hypothesis that NRF1 activates aggrephagy to compensate for proteasome dysfunction.

### Genome-wide transcriptome analyses leads to the identification of autophagy-related genes as NRF1-targeted genes after proteasome impairment

**Figure 2:**
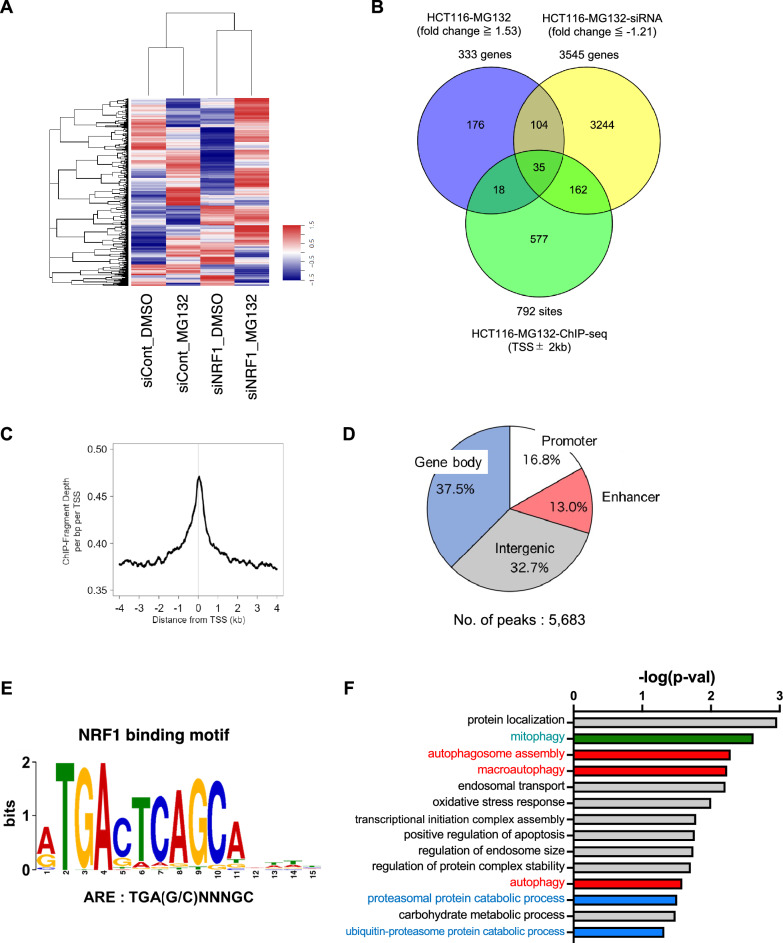
Genome-wide transcriptome analysis reveals the biological implication of NRF1 in autophagy during proteasome inhibition. (**A**) A heatmap illustrating the altered expression of genes in HCT116 cells after treatment with MG132 (1 µM) for 16 h and/or *NRF1* knockdown. (**B**) Integrated analysis using DNA microarray and chromatin immunoprecipitation-sequencing (ChIP-seq) data to identify genes directly targeted by NRF1. DNA microarray analysis revealed 333 upregulated genes (≥ 1.53-fold) in HCT116 cells treated with MG132 (HCT116-MG132), and among these genes, 139 genes were found to be significantly downregulated by concomitant siNRF1 transfection (≤ − 1.21-fold) (HCT116-MG132-siNRF1). ChIP-seq analysis revealed 792 sites recognized by NRF1 within ± 2 kbp of the transcriptional start site (TSS ± 2 kbp) after 1 µM MG132 treatment for 16 h (HCT116-MG132-ChIP-seq). Based on these results, 35 genes were identified as NRF1 target gene candidates. The results of these analyses are presented in a Venn diagram. (**C**) The profile of the ChIP-seq signals of NRF1 around the TSS. (**D**) Distribution profiles of NRF1-binding regions on the genome as identified via ChIP-seq analysis (promoter: transcription start site ± 3 kb, enhancer: transcription start site − 20 kb ~  − 3 kb, gene body: transcription start site + 3 kb ~ transcription termination site + 1 kb, intergenic: other regions in the genome. (**E**) NRF1-binding motifs identified using MEME-ChIP from the results of the ChIP-seq analysis. (**F**) DAVID software was used for a Gene Ontology analysis of the 35 most commonly identified genes (Table S3)^46^.

To investigate the possibility of NRF1-driven autophagy further, genome-wide transcriptome analyses using DNA microarray and ChIP-sequencing (ChIP-seq) data were performed to identify direct target genes of NRF1 after proteasome inhibition (Fig. 2). Initially, we carried out DNA microarray analysis using HCT116 cells treated with MG132 and identified the upregulation of 333 genes after proteasome inhibition (fold change ≥ 1.53) (Fig. 2A,B). Among these genes, 139 genes showed significantly decreased expression after siNRF1 transfection (fold change ≤ − 1.21), indicating that NRF1 regulated the expression of these genes (Table S1).

Next, to identify the NRF1-binding region in the genome, we performed ChIP-seq analysis using HCT116 cells treated with MG132. ChIP DNA samples were prepared using an anti-Nrf1 antibody^33^ and subjected to deep sequencing. The NRF1 ChIP fragment was found to be highly enriched around transcription start sites (TSSs) (Fig. 2C). A total of 792 sites within ± 2 kbp of a TSS in the ChIP DNA library were shown to be significantly enriched (Fig. 2B; Table S2). The NRF1-binding sites were abundant in the gene body and intergenic regions and to a slightly lesser extent in enhancer and promoter regions (Fig. 2D). Importantly, a de novo motif analysis indicated that the enriched sites carried the consensus ARE motif (TGA(G/C)NNNGC), which is recognized by NRF1 ref, suggesting that the NRF1-binding regions had been successfully identified (Fig. 2E).

Finally, integrated analyses using omics data obtained through the DNA microarray and ChIP-seq analyses led to the identification of 35 candidate NRF1 targeted genes after proteasome dysfunction (Fig. 2B; Table S3). As expected, a Gene Ontology analysis revealed that certain genes were associated with autophagosome assembly, macroautophagy, and autophagy as well as proteasomal protein catabolic processes and ubiquitin‒proteasome protein catabolic processes (Fig. 2F; Table S3). Altogether, these results provide compelling evidence indicating that NRF1 is critical for inducing autophagy after proteasome inhibition.

### NRF1 regulates the expression of autophagy-related genes after proteasome inhibition

**Figure 3:**
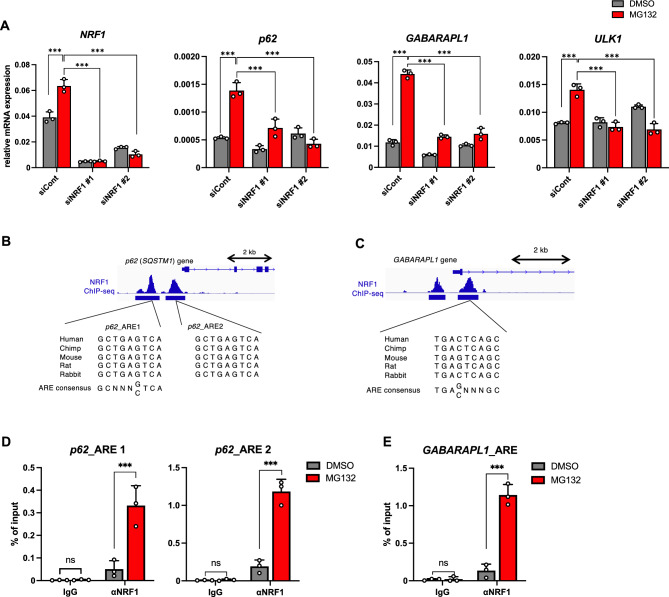
NRF1 directly induces the expression of autophagy-related *p62* and *GABARAPL1* genes after proteasome inhibition. (**A**) Two days after siRNA transfection, HCT116 cells were treated with DMSO or MG132 (1 μM) for 16 h, and then, the mRNA levels of autophagy-related genes were measured by RT‒qPCR (n = 3). The expression levels were normalized to the *β-actin* levels. (**B**–**E**) The recruitment of NRF1 to the promoters of the *p62* and *GABARAPL1* genes in HCT116 cells after proteasome inhibition was determined by ChIP-seq (**B** and **C**) and ChIP analyses (**D** and **E**). ChIP-seq signals and NRF1 peaks are shown on the genome loci of *p62* and *GABARAPL1* in the human genome using Integrative Genomics Viewer (IGV). In **D** and **E**, HCT116 cells were treated with MG132 (1 μM) for 16 h and then subjected to ChIP‒qPCR analysis using an anti-Nrf1 antibody (n = 3). (**A**, **D**, and **E**) ANOVA followed by Tukey’s test, mean ± SD, ***p < 0.005, ns: not significant.

Among the autophagy-related genes identified, we focused on *p62/Sequestosome 1* (*SQSTM1*), *GABA(A) receptor-associated protein like 1* (*GABARAPL1*), and *Unc-51-like autophagy activating kinase 1* (*ULK1*) (Table S3). To validate the results of transcriptome analyses that NRF1 would directly modulates the expression of these genes, we knocked down *NRF1* and performed an RT‒qPCR analysis using HCT116 cells and observed that NRF1 activated *p62*, *GABARAPL1* and *ULK1* expression in response to proteasome inhibition (Fig. 3A). The genome databases indicated that the *p62*, *GABARAPL1* and *ULK1* genes carried species-conserved ARE sites, and our ChIP data revealed that these sites were directly recognized by NRF1 (Fig. 3B–E; Fig. S1). These results further strengthen the argument that NRF1 plays biological roles in the regulatory mechanisms underlying autophagy. Because no significant effect of NRF1 on ULK1 protein levels was observed (Fig. S1), we directed our attention toward p62 and GABARAPL1.

### NRF1 activates aggrephagy, a type of selective autophagy, by inducing p62 gene expression

**Figure 4:**
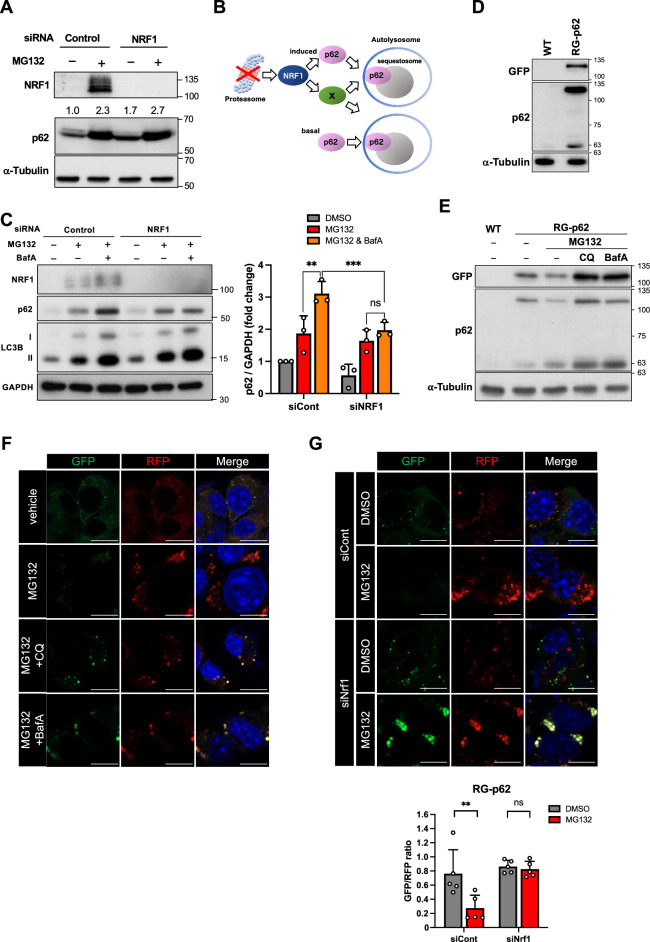
NRF1 leads to the activation of aggrephagy by inducing *p62* expression. (**A**) *NRF1* knockdown did not decrease the p62 protein levels that had accumulated after MG132 treatment. Two days after siRNA transfection, HCT116 cells were treated with 1 μM MG132 for 16 h, and p62 protein levels were measured by immunoblotting. A representative result from three independent experiments is shown. The values indicate relative band intensities of p62. (**B**) A hypothetical model of the simultaneous activation of p62 expression and autophagy induced by NRF1. (**C**) NRF1 augmented the autophagic degradation of p62. Two days after siRNA transfection, HCT116 cells were treated with MG132 (1 μM) and/or BafA (10 nM) for 16 h. The levels of p62 protein were quantified by ImageJ and normalized to GAPDH expression levels (n = 3). (**D**–**F**) Generation of the p62 reporter RG-p62, which is a fusion protein comprising full-length p62 protein with red and green fluorescent proteins (RFP and GFP, respectively), to monitor autophagic proteolysis in the cells. As shown in (**D**), the expression of RG-p62 in Neuro2a cells was determined by immunoblot analysis using the antibodies identified in the figures. An immune-positive band, evident with an anti-p62 antibody (63 kDa), was identified as the endogenous p62 protein in Neuro2a cells, which was stabilized by exogenous RG-p62 expression. As shown in (**E**) and (**F**), the RG-p62 reporter protein recapitulated lysosomal proteolysis of endogenous p62 proteins. Neuro2a cells stably expressing RG-p62 were treated with MG132 (1 μM) in combination with chloroquine (CQ) (20 µM) or BafA (10 nM) for 16 h, and whole-cell extracts were then subjected to immunoblot analysis. As shown in (**F**), the fluorescence of GFP and RFP emitted by RG-p62 was visualized using a confocal microscope. The nuclei were stained with DAPI. (**G**) NRF1 promoted the clearance of p62-formed aggregates via autophagy after proteasome inhibition. The fluorescence of GFP and RFP in over 100 cells (five different views) was monitored as described in (**F**), and the GFP/RFP ratios are shown in the below graph. Scale bar, 10 μm. (**C**, **G**) ANOVA followed by Tukey’s test: mean ± SD, **p < 0.01, ***p < 0.005, ns: not significant.

We first examined the NRF1–p62 axis and analyzed its roles in proteostasis. To evaluate whether NRF1 regulates p62 expression at the protein level, we performed immunoblot analyses (Fig. 4A). MG132 treatment significantly increased the levels of the p62 protein as well as the NRF1 protein, supporting our findings showing that NRF1 mediated the expression of the *p62* gene (Fig. 3A,B,D). Surprisingly, however, *NRF1* knockdown failed to decrease the p62 protein level; in contrast, it rather led to a slight increase in protein expression (Fig. 4A). This unexpected result led us to hypothesize that knocking down *NRF1* represses not only *p62* transcription but also autophagic proteolysis of p62, resulting in a slight accumulation of p62 proteins due to competition between these two opposing effects (Fig. 4B). To test this hypothesis, we examined the impact of BafA treatment on the amount of p62 protein accumulation after treatment with MG132 (Fig. 4C). BafA treatment significantly increased the levels of the LC3-II protein, implying that autophagy was suppressed. Under these experimental conditions, BafA treatment led to greater p62 protein accumulation than MG132 treatment alone; however, we did not observe an increase in p62 protein levels induced by BafA in combination with *NRF1* knockdown. These findings strongly suggested that NRF1 promotes autophagic degradation of p62 after proteasome inhibition.

To further assess whether NRF1 is involved in the activation of aggrephagy and thus degrades p62 puncta, we generated a p62 reporter protein, RG-p62, to monitor the autophagic proteolysis of p62 (Fig. 4D). This reporter protein consists of full-length p62 fused with red fluorescent protein (RFP) and green fluorescent protein (GFP), and it was stably expressed in mouse neuroblastoma Neuro2A cells. Under normal conditions, the RG-p62 reporter formed small puncta that emitted both RFP and GFP fluorescence in the cytoplasm (Fig. 4E,F). After proteasome inhibition and aggrephagy activation mediated by MG132 treatment, this reporter was found in larger condensates and emitted only RFP fluorescence, suggesting that these puncta had fused with lysosomes (i.e., autolysosomes), and that the acidic conditions in lysosomes weakened the GFP fluorescence signal emitted by RG-p62. Furthermore, cotreatment with BafA or chloroquine (CQ) resulted in the recovery of GFP florescence in p62 puncta, supporting the idea that the reporter underwent lysosomal proteolysis. Considering these observations, we concluded that the RG-p62 reporter protein recapitulated autophagic proteolysis of endogenous p62 proteins (Fig. 4E; 63 kDa). Next, using this p62 reporter system, we examined the effects of *Nrf1* knockdown on aggrephagy-mediated p62 degradation by quantifying the fluorescence ratio of GFP to RFP in p62-mediated puncta that had formed within cells (Fig. 4G). As expected, *Nrf1* knockdown did not result in a decreased GFP/RFP fluorescence ratio after proteasome inhibition, implying that Nrf1 enhanced the autophagic degradation of the p62 puncta. Collectively, these data demonstrate that NRF1 activates not only the transcription of *p62* but also the proteolysis of p62-positive puncta via aggrephagy after proteasome suppression.

### NRF1 is required for the formation of p62-positive puncta after proteasome inhibition

**Figure 5:**
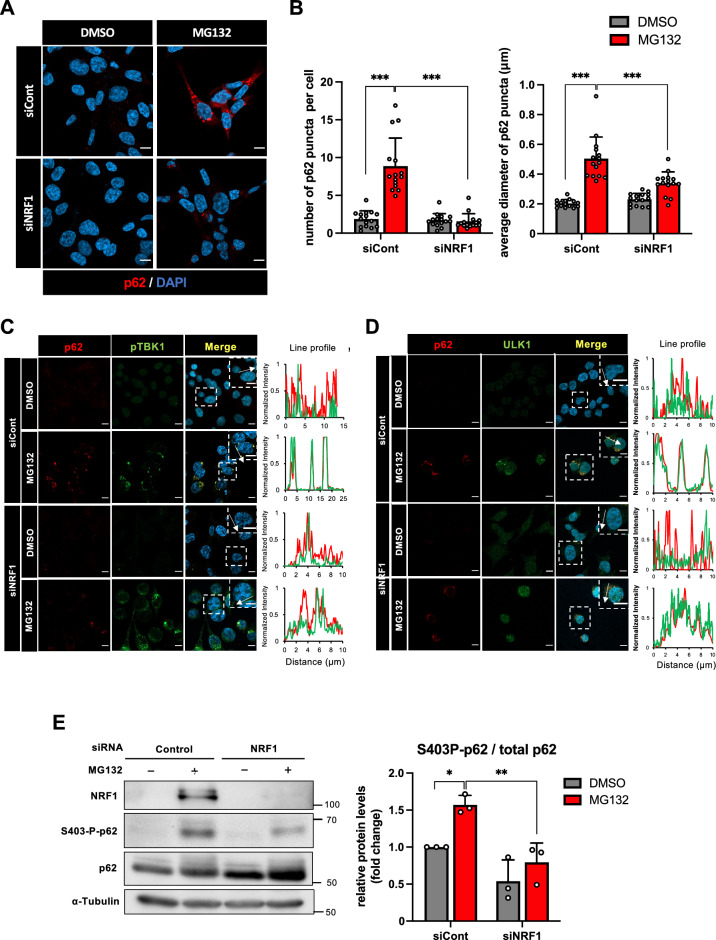
NRF1 plays a critical role in the formation of p62-positive puncta induced by proteasome inhibition. (**A** and **B**) Two days after siRNA transfection, HCT116 cells were treated with MG132 (1 μM) for 16 h, p62-positive puncta were visualized by immunostaining, and fluorescence images were acquired with a confocal laser microscope. As shown in (**B**), the number per cell and the median diameter of puncta in more than 400 cells (15 different views) were analyzed using ImageJ. (**C**, **D**) Colocalization of p62 (red) with phosphorylated TBK1 (p-TBK1, green) or ULK1 (green). Representative results from three different experiments are shown. Cell treatments and immunostaining were performed as described in (**A**). Line profiles demonstrate fluorescence intensities derived from p62 (red), p-TBK1 (green) and ULK1 (green) in representative cells depicted by white squares. Scale bar 10 μm. (**E**) NRF1 promoted the phosphorylation of Ser403 in p62 proteins. Cell treatments and immunostaining were carried out as described in (**A**) (n = 3). (**B**, **E**) ANOVA followed by Tukey’s test: mean ± SD, *p < 0.05, **p < 0.01, ***p < 0.005.

Given that proteasome inhibition induced the formation of p62 puncta^1, 3, 34^, we next investigated the involvement of NRF1 in this process. While MG132 treatment markedly increased the formation rate of p62 puncta in cells, surprisingly, simultaneous *NRF1* knockdown substantially mitigated the effects of proteasome inhibition on both the number and size of the puncta (Fig. 5A,B). Importantly, as shown in Fig. 4A,C, p62 proteins were present in *NRF1*-knockdown cells without forming the puncta. These findings provide evidence showing that NRF1 plays a critical role in the formation of p62-positive puncta after proteasome suppression.

p62 is phosphorylated by several kinases, such as TBK1 and ULK1, leading to increased phase separation-driven sequestosome formation. In this context, phosphorylation of TBK1 itself is essential for its activation. To elucidate the molecular mechanisms underlying NRF1-driven p62 punctate formation, we explored whether p62 colocalized with phosphorylated TBK1 and ULK1 on the puncta. Supporting our hypothesis, immunohistochemical staining revealed that they colocalized with the puncta, and this effect was abrogated by *NRF1* knockdown because p62-positive puncta disappeared (Fig. 5C,D). Considering these observations, we surmised that ULK1 and/or TBK1 may phosphorylate Ser403 in p62, facilitating punctate formation. To test this hypothesis, we examined the effects of *NRF1* knockdown on the phosphorylation of p62 and found that decreased NRF1 levels significantly attenuated the phosphorylation of p62 Ser403 (Fig. 5E). Moreover, we made extensive efforts to elucidate the underlying mechanism of NRF1-driven p62-positive punctate formation mediated through phosphorylation; however, we were unable to fully identify this mechanism, and therefore, further examination is required. These results demonstrate that NRF1 is required for the formation of p62-positive puncta after proteasome inhibition.

### NRF1 stimulates GABARAPL1 expression during aggrephagy

We next investigated the NRF1–GABARAPL1 axis and its role in proteostasis. First, to assess the selective induction of GABARAPL1 by NRF1 compared to that of the six other members of the ATG8 family, we conducted knockdown experiments (Fig. S2). Proteasome inhibition induced the expression of *MAP1LC3B* and *MAP1LC3C*, albeit to a lesser extent than *GABARAPL1*. However, the expression of these genes was not significantly reduced by *NRF1* knockdown, implying that NRF1 specifically controlled *GABARAPL1* expression but not the other ATG8 family member genes after proteasome dysfunction.

**Figure 6:**
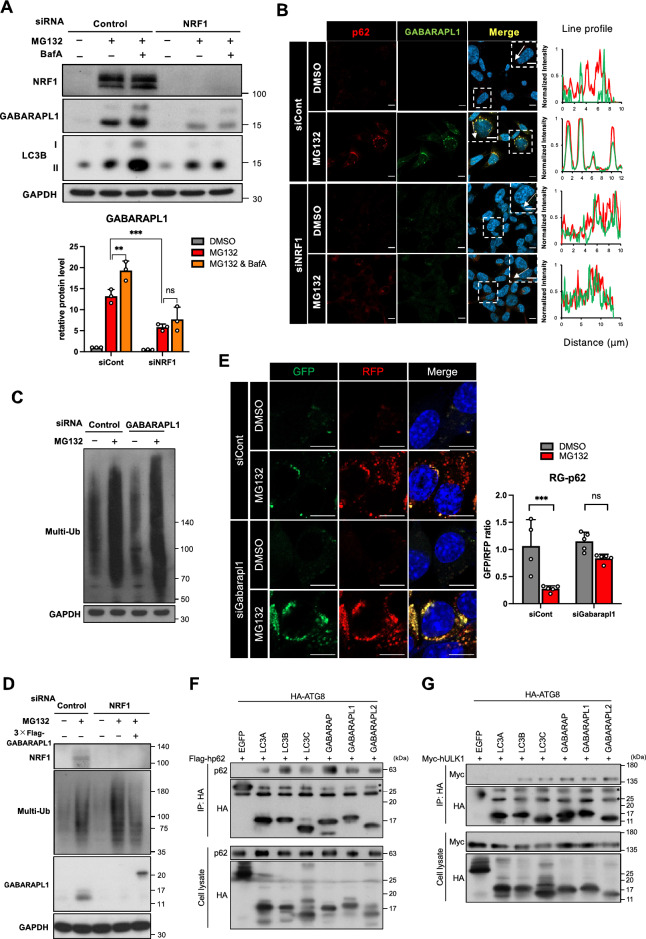
NRF1 leads to the induction of GABARAPL1 expression for aggrephagy. (**A**) *NRF1* knockdown substantially reduced the expression of GABARAPL1 proteins. Cell treatment and immunoblotting were conducted as described in the legend of Fig. 4A. The lower graph represents the quantified protein levels of GABARAPL1 normalized to the GAPDH (n = 3). (**B**) Colocalization of p62 with GABARAPL1 in response to proteasome repression. Cell treatments and immunostaining were carried out as described in the legend of Fig. 5A. Representative results from three different experiments are shown. Line profiles demonstrate fluorescence intensities derived from p62 (red) and GABARAPL1 (green) in representative cells depicted by white squares. Scale bar, 10 μm. (**C** and **D**) GABARAPL1 facilitated the removal of Ub-proteins during proteasome inhibition. Two days after transfection of *GABARAPL1* siRNA, HCT116 cells were treated with MG132 (1 μM) for 16 h. Representative results from three-independent experiments are shown. As shown in (**D**), for the rescue experiment, the 3xFlag-GABARAPL1 expression vector was cotransfected along with *NRF1* siRNA. (**E**) *GABARAPL1* knockdown reduced autophagic proteolysis of the p62 reporter RG-p62 after proteasome inhibition. Cell treatments and fluorescence imaging (58 cells (siCont_DMSO) and more than 100 cells (other conditions)) were conducted as described in the legend of Fig. 4F,G. (**F** and **G**) Association of GABARAPL1 with p62 and ULK1 as shown in an immunoprecipitation analysis. The expression plasmids indicated in the figures were transfected into 293T cells. Twenty-four hours after transfection, the cells were treated with MG132 (1 µM) for 16 h. Whole-cell extracts (Cell lysate) were prepared and subjected to immunoprecipitation (IP) using anti-HA antibodies and immunoblot analysis. The HA-fused enhanced green fluorescent protein (HA-EGFP) plasmid was utilized as a negative control for the HA-fused ATG8 protein plasmids. Representative results from three-independent experiments are shown. Asterisks indicate immunopositive bands derived from immunogloblin light chains and non-specific proteins. (**A** and **E**) ANOVA followed by Tukey’s test: mean ± SD, **p < 0.01, ***p < 0.005, ns: not significant.

We sought to determine whether NRF1 upregulates the expression of GABARAPL1 protein. Similar to the results for p62 (Fig. 4C), the results obtained after MG132 treatment indicated a markedly increase in the amount of GABARAPL1 protein, and the protein levels were further increased after cotreatment with BafA, indicating that GABARAPL1 was also degraded by lysosomes (Fig. 6A). This induction of GABARAPL1 protein expression mediated by MG132 treatment was substantially diminished after *NRF1* knockdown, implying that GABARAPL1 protein expression was mediated by NRF1. By immunohistochemical staining, we also confirmed the colocalization of GABARAPL1 with p62-positive puncta after MG132 treatment (Fig. 6B). These results demonstrate that proteasome inhibition leads to an increase in GABARAPL1 protein levels during aggrephagy and that the increase was NRF1 dependent.

Furthermore, to investigate the significance of the NRF1–GABARAPL1 axis in proteostasis, we measured the Ub-protein levels in cells after *GABARAPL1* knockdown following MG132 treatment. As expected, *GABARAPL1* knockdown substantially impaired the clearance of Ub-proteins in these cells (Fig. 6C). Additionally, overexpression of GABARAPL1 rescued the clearance of Ub-proteins that had accumulated in *NRF1*-knockdown cells (Fig. 6D). Moreover, using the RG-p62 reporter, we demonstrated that Gabarapl1 played a crucial role in the autophagic degradation of p62 puncta after proteasome inhibition (Fig. 6E). These observations highlight the indispensable roles played by the NRF1–GABARAPL1 axis in aggrephagy for the maintenance of proteostasis.

To investigate the biological significance of the selective induction of GABARAPL1 mediated by NRF1, we explored the binding affinity of GABARAPL1 for p62 and ULK1 and compared it to that of other ATG8 family proteins via immunoprecipitation. The interaction of GABARAPL1 with p62 was observed (Fig. 6F). Similarly, GABARAPL1 was found to be associated with ULK1 (Fig. 6G), supporting a previous report^31^. These results substantiate the biological function of the NRF1-GABARAPL1 axis in aggrephagy. Altogether, our current findings provide evidence for the involvement of the NRF1–GABARAPL1 axis in aggrephagy and with p62 after proteasome inhibition.

### NRF1-related factor NRF2 also upregulates the expression of p62 and GABARPL1 during proteasome inhibition

**Figure 7:**
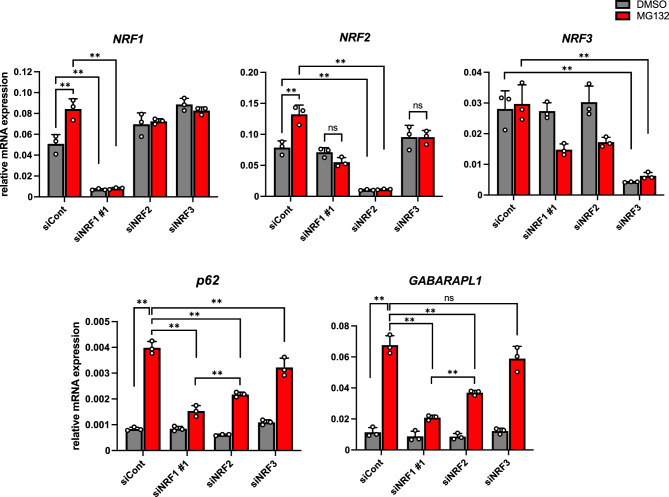
Cooperative regulation of *p62* and *GABARAPL1* gene expression by NRF1 and NRF2. Two days after siRNA transfection, HCT116 cells were treated with DMSO or MG132 (1 μM) for 16 h, and then, the mRNA levels of *NRF1-3* and autophagy-related genes were measured by RT‒qPCR (n = 3). The expression levels were normalized to the *β-actin* levels. ANOVA followed by Tukey’s test: mean ± SD, **p < 0.01, ns: not significant.

NRF2 has been established to play a crucial and positive role in regulating autophagy by inducing p62^35^. To investigate the biological relevance of NRF2 and another NRF1-related factor NRF3 in aggrephagy activation under proteasome dysfunction, we conducted siRNA and RT-qPCR analyses utilizing *NRF2* and *NRF3*-targeting siRNAs (Fig. 7). Indeed, *NRF2* knockdown also resulted in the reduction of *p62* and *GABARAPL1* induction by MG132 treatment, albeit to a slightly lesser extent than observed with *NRF*1 knockdown, implying that NRF1 plays a more substantial role in the induced expression of these genes than NRF2. Consistent with this notion, *NRF1* knockdown reduced the induced expression of *NRF2* upon MG132 treatment, indicating that NRF1 also participates in the activation of *NRF2* expression and, consequently, its autophagy activation. Finally, our results indicated that the involvement of NRF3 in the gene regulation of these genes appears to be weak. Collectively, these data suggest the possibility of cooperative gene regulation of *p62* and *GABARAPL1* by NRF1 and NRF2 in aggrephagy.

## Discussion

**Figure 8:**
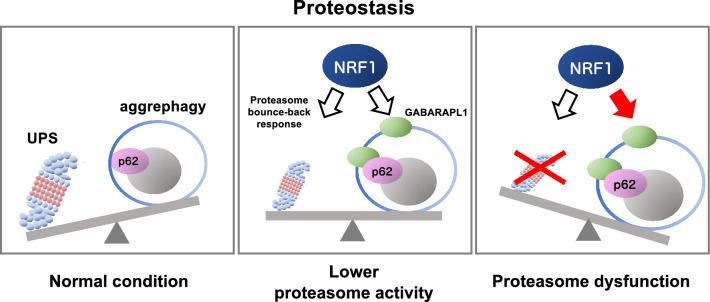
Schematic representation of NRF1-mediated activation of aggrephagy mediated by impaired proteasome activity. The ubiquitin‒proteasome system (UPS) and autophagy are protein degradation pathways essential for maintaining proteostasis (left panel). When proteasome activity is reduced, due to several reasons such as chemical inhibitors and aging, the transcription factor NRF1 is activated, leading to upregulation of proteasome gene expression (i.e., proteasome bounce-back response) (middle panel)^7–9^. Furthermore, complete proteasome dysfunction activates NRF1-mediated aggrephagy, inducing the expression of aggrephagy-related *p62* and *GABARAPL1* genes (right). These cellular responses enable survival against proteasome dysfunction by maintaining proteostasis.

In this study, we analyzed the cooperative mechanisms of the ubiquitin–proteasome system (UPS) and autophagy in the maintenance of proteostasis and subsequently revealed that the transcription factor NRF1 activated aggrephagy to facilitate the elimination of Ub-proteins by inducing the expression of *p62* and *GABARAPL1* in response to proteasome inhibition. Notably, given that NRF1 complements proteasome inactivation via the induction of proteasome gene expression (i.e., proteasome bounce-back response)^7–9^, these findings provide new insights into the mechanisms underlying proteostasis maintenance: i.e., the upregulation of two major proteolysis systems, the UPS and aggrephagy, regulated by NRF1 in response to proteasome dysfunction (Fig. 8). It has been widely recognized that disruption of proteostasis, resulting from various factors, including aging and exposure to chemical inhibitors, contributes to a range of human diseases, such as neurodegenerative diseases and cancer. In contrast, the maintenance mechanisms underlying proteostasis confer resistance to the therapeutics designed to target the proteasome, such as bortezomib, which is utilized to treat multiple myeloma. Therefore, our findings suggest that positive or negative regulation of NRF1 activity through control may represent a novel therapeutic approach for overcoming these diseases and therapeutic resistance, as discussed below.

We discovered that NRF1 was also involved in the formation of p62-positive puncta after proteasome activity was reduced (Fig. 5). The underlying mechanism for p62-positive aggresome formation was revealed: p62 is phosphorylated by ULK1 and TBK1 and subsequently binds to Ub-proteins, leading to the formation of p62-liquid droplets (or p62-bodies) through phase separation and ultimately to p62-positive punctate formation^23–28^. Consistent with these findings, we observed that *NRF1* knockdown significantly reduced the phosphorylation levels of p62 at Ser403, which led to punctate formation (Fig. 5E). We have made extensive efforts to elucidate the underlying mechanism by which NRF1 drives the formation of p62 puncta; however, we were unable to fully identify the mechanism. Therefore, we are currently studying the mechanism in our ongoing project.

One of the important arguments that guided this research suggests the biological significance of the selective induction of GABARAPL1, in contrast to ATG8 family members, mediated by NRF1 for protein quality control (Fig. 6; Fig. S2). We demonstrated that GABARAPL1 displayed comparable binding affinity for both p62 and ULK1 compared to other LC3 subfamily proteins (Fig. 6F,G). This result reveals that GABARAPL1 would participate in aggrephagy along with these factors, but it may also indicate that GABARAPL1 is not selectively engaged in this process among ATG8 family proteins. Alternatively, the NRF1-GABARAPL1 axis could potentially play a role in the activation of lysosome fusion to autophagosomes. The reason for this is that GABARAPL1 harbors the GABARAP interaction motif (GIM), a specific motif in the LC3-interacting region (LIR)^21, 36^. The GIM enables GABARAP subfamily proteins to closely associate with PLEKHM1, a protein that mediates autophagosome-lysosome fusion through interaction with HOPS^21, 37^. Furthermore, GABARAPL1 has been shown to promote autophagosome closure^32^. Although the meaning of the selective upregulation of GABARAPL1 mediated by NRF1 remains unclear, our findings suggest that the NRF1–GABARAPL1 axis plays a crucial role in aggrephagy and leads to the efficient clearance of protein aggregates.

Our findings have revealed the regulatory transcriptional mechanisms that underlie aggrephagy. Classical autophagy induced in response to nutrient starvation is mediated by posttranslational modifications such as phosphorylation and protein processing, allowing for rapid cellular responses that ensure cell survival against stress. Aggrephagy may not always lead to this immediate and acute response and, in contrast, may selectively degrade substrates mediated through transcriptional regulation process, which is a time-consuming process. Hence, it appears that transcriptional regulation contributes to the diversity and complexity of autophagy. NRF1-related proteins have been implicated in the regulation of autophagy in various biological events because several autophagy-related genes carry ARE sites recognized by these proteins^38^. For instance, we have recently demonstrated that NRF3 coordinates the melanogenesis cascade by activating the expression of autophagy-related genes such as *GABARAPL2* and *ULK2*, which are required for the formation of autophagosomes^39^. NRF2 also modulates the expression of autophagy-related genes to eliminate protein aggregates^38^. Intriguingly, after autophagy dysfunction, accumulated p62 proteins activated NRF2 by competitively binding to the NRF2 repressor KEAP1^40, 41^. Furthermore, in certain cancer cells, NRF2 induced the expression of proteasome genes after autophagy repression, thereby adapting to autophagy dysfunction^42^. These findings indicate a possible mechanism by which NRF2 plays a role opposite to that implicated by our findings, i.e., proteasome activity is induced in response to autophagy inhibition, which has rarely been reported^1^.

As mentioned above, NRF1 is considered a promising drug target because of its ability to activate both the UPS and aggrephagy simultaneously. Under physiological conditions, the biological function of NRF1 is suppressed by sequestration in the endoplasmic reticulum (ER) and by proteasomal degradation of NRF1 via the ERAD E3 ubiquitin ligase HRD1^7–9^. Proteasome dysfunction leads to the activation of NRF1 by releasing it from these repression mechanisms, subsequently inducing the expression of proteasome genes and aggrephagy-related *p62* and *GABARAPL1* genes. Based on these insights, it is plausible to consider that NRF1 activators may be potentially used to treat certain diseases associated with proteostasis dysfunction, including neurodegeneration and metabolic diseases, by augmenting the two protein quality control systems. In contrast, it is also conceivable that NRF1 activation may confer resistance to bortezomib, a proteasome inhibitor commonly utilized in the treatment of multiple myeloma. In this regard, it has been reported that HIV protease inhibitors such as nelfinavir suppressed NRF1-driven resistance to bortezomib^14–18^ because these reagents repressed the protease activity of DDI2, an NRF1 activator that carries a protease domain similar to that of HIV proteases. Hence, we assume that targeting NRF1 through both positive and negative strategies is a promising approach to developing therapies for diseases caused by proteostasis dysfunction.

Finally, we would like to discuss the distinctions in our research from the study published by Sha Z. et al. that is relevant to our investigation^43^. Our findings differed from their results in the following ways. First, we demonstrated the biological relevance between NRF1 and aggrephagy through genome-wide transcriptome analysis (Fig. 2). To our knowledge, this is the first report of an integrated analysis of transcriptome and ChIP-seq data performed to examine endogenous human NRF1 protein functions. Although several studies have employed similar analyses using NRF1-overexpressing cell lines^44, 45^, we observed differences in NRF1 target genes between our results and the previous reports, likely attributable to variations in experimental conditions, such as NRF1 expression levels and cell types. Second, through a ChIP analysis, we discovered that NRF1 directly modulated the expression of *GABARAPL1*, which harbors a species-conserved ARE site that is recognized by NRF1 family proteins (Figs. 3 and 6). Third, we developed a p62 flux reporter, enabling us to monitor its proteolysis mediated by aggrephagy (Fig. 4D–G). Finally, and most importantly, we discovered that NRF1 is involved in the mechanisms of p62-positive punctate formation (Fig. 5). We have also revealed that knockdown of *NRF1* significantly reduced the phosphorylation levels of Ser403 in p62. The phosphorylation of p62 has been previously reported to be essential for the formation of p62-puncta because it enhances p62 binding to Ub-proteins and then to subsequent phase separation^22–25^. Further investigation is needed to fully elucidate the mechanism.

## Methods

### Reagents and antibodies

In this study, we utilized MG132 (Z-Leu-Leu-Leu-H (aldehyde), Peptide Institute), bafilomycin A1 (Sigma), and chloroquine (Wako). The antibodies utilized in this study were anti-NRF1 (D5B10; Cell Signaling Technology), anti-p62 (PM045; MBL), anti-S403-P-p62 (4F6; MBL), anti-ULK1 (D8H5; Cell Signaling Technology), anti-TBK1 (ab109735; Abcam), anti-GFP (sc-9996; Santa Cruz), anti-LC3B (L7543; Sigma), anti-GAPDH (6C5; Santa Cruz), anti-α-tubulin (DM1A; Sigma), anti-HA (12CA5; Sigma), and anti-Myc (sc-40; Santa Cruz) for immunoblot analyses; anti-p62 (PM066; MBL), anti-ULK1 (F-4; Santa Cruz), and anti-S172-P-TBK1 (D52C2; Cell Signaling Technology) for immunofluorescence analysis; anti-ubiquitin (clone FK2) (D058-3; MBL), anti-GABARAPL1 (D5R9Y; Cell Signaling Technology) for immunofluorescence and immunoblot analyses, anti-HA (3F10; Sigma) for immunoprecipitation; and normal rabbit anti-IgG antibody (Wako) and rabbit anti-Nrf1 polyclonal antibody (raised against mouse NRF1 residues from 292 to 741)^33^ for ChIP assay.

### Cell cultures

Human colorectal cancer HCT116 cells, human embryonic kidney 293T cells and mouse neuroblastoma Neuro2a cells were purchased from the RIKEN Bioresource Research Center, Japan. These cell lines and their derivative cell lines were cultured in DMEM (Wako Pure Chemicals) supplemented with 10% FBS (Sigma‒Aldrich) and 1% penicillin/streptomycin (Wako Pure Chemicals). All cell lines were cultured in a humidified incubator with 5% CO_2_ at 37 °C.

### siRNA transfection

Transfection of short interfering RNA (siRNA) was performed using RNAiMAX (Thermo Fisher Scientific.) according to the manufacturer’s instructions. The sequences of the siRNAs utilized in this study are listed in Table S4.

### RNA extraction and real-time quantitative PCR (RT‒qPCR)

Total RNA was prepared using ISOGENII (Nippon Gene). One microgram of total RNA was synthesized into cDNA using random hexamer primers (Takara Bio) and Moloney murine leukemia virus (M-MLV) reverse transcriptase (Thermo Fisher Scientific.). RT‒qPCR was performed using TB Green Premix Ex Taq II (Takara Bio) and a Thermal Cycler Dice Real Time System II (Takara Bio). The PCR conditions were 95 °C for 30 s, 30 cycles of 95 °C for 5 s, 60 °C for 30 s and 95 °C for 15 s, and 60 °C for 30 s and 95 °C for 15 s. All target gene expression levels were normalized to *β-actin* or *GAPDH* expression. The sequences of the primers used in this study are listed in Table S4.

### Preparation of whole-cell extracts and immunoblot analysis

Whole-cell extracts of cells treated with the reagents indicated in the figures were prepared by lysing cells in SDS sample buffer (50 mM Tris–HCl (pH 6.8), 10% glycerol and 1% SDS). The protein quantities in the cell extracts were measured with a bicinchoninic acid (BCA) kit (Wako Pure Chemicals). Proteins were separated by sodium dodecyl sulfate‒polyacrylamide gel electrophoresis (SDS‒PAGE) and transferred to PVDF membranes (Immobilon-P transfer membranes, Millipore). The blots were treated with the primary antibodies indicated in the figures and with the corresponding horseradish peroxidase-conjugated secondary antibody (Thermo Fisher Scientific). The protein bands were visualized using enhanced chemiluminescence (GE Healthcare).

### Proteasome activity assay

After treating the cells with the reagents under the conditions indicated in the figures, whole-cell extracts were prepared in lysis buffer (25 mM Tris–HCl [pH 7.5], 50 mM MgCl_2_, 2% NP-40, and 1 mM DTT). The protein quantities in the cell extracts were measured with a bicinchoninic acid (BCA) kit (Wako Pure Chemicals). Proteasome activity was measured using 10 µg of protein mixed with 2 mM ATP and 50 µM fluorogenic peptide substrate Suc-LLVY-AMC (succinyl-Leu-Leu-Val-Tyr-7-amino-4-methylcoumarin; Peptide Institute). Fluorescence was measured on a microplate fluorometer (Synergy HTX; Bio Tek Instruments) every 5 min for 1 h (380-nm excitation, 460-nm emission). Proteasome activity was calculated as the fluorescence intensity change over time using the Microsoft Excel slope function.

### DNA microarray analysis

HCT116 cells were treated with MG132 (1 µM) for 16 h and then subjected to RNA preparation. Total RNA was processed with an Ambion WT expression kit (Affymetrix) in accordance with the manufacturer’s instructions. cRNA was fragmented, labeled, and hybridized to the Affymetrix human gene 1.0 ST arrays using a GeneChip WT terminal labeling and hybridization kit (Affymetrix). GeneChip Fluidics Statin 450 was used for processing of the arrays, and fluorescence signals were detected with a GeneChip scanner 3000-7 G. Images were analyzed with the GeneChip operating software (Affymetrix). Finally, the Expression console and Transcription analysis console (Affymetrix) were used to analyze the data. The DAVID functional annotation tool was used for the GO analysis of the biological process terms^46^. The DNA microarray data have been deposited in the Gene Expression Omnibus database (accession number GSE227232) and are presented in Table S1.

### Chromatin immunoprecipitation (ChIP) and ChIP sequencing (ChIP-seq) analyses

After treating cells with the reagents indicated in the figures, the cells were fixed in 1% formaldehyde for 10 min, followed by quenching with 125 mM glycine and two washes with phosphate-buffered saline (PBS). The cells were lysed in cell lysis buffer (5 mM Tris–HCl [pH 8.0], 85 mM KCl, 0.5% NP-40 and protease inhibitor cocktail (Nacalai Tesque)) and then centrifuged at 2000 rpm for 3 min at 4 °C. The pellets were then lysed with nuclei lysis buffer (50 mM Tris–HCl [pH 8.0], 10 mM EDTA, 1% SDS and protease inhibitor cocktail), followed by sonication using a Bioruptor (Tosho Electric). After centrifugation at 15,000 rpm for 10 min at 8 °C, the supernatants were collected. The supernatants were diluted with ChIP dilution buffer (16.7 mM Tris–HCl [pH 8.0], 167 mM NaCl, 1.2 mM EDTA, 0.01% SDS, 1.1% Triton X-100 and protease inhibitor cocktail), and proteins were immunoprecipitated using the antibodies indicated in the figures and Dynabeads protein G (Thermo Fisher Scientific). The beads were washed with low-salt wash buffer (20 mM Tris–HCl [pH 8.0], 150 mM NaCl, 2 mM EDTA, 0.1% SDS and 1% Triton X-100), high-salt wash buffer (20 mM Tris–HCl [pH 8.0], 500 mM NaCl, 2 mM EDTA, 0.1% SDS, and 1% Triton X-100), lithium wash buffer (10 mM Tris–HCl [pH 8.0], 250 mM LiCl, 1% sodium deoxycholate, 1 mM EDTA and 1% NP-40) and Tris–EDTA (TE) buffer. Cross-linking was reversed overnight at 65 °C in ChIP elution buffer (1% SDS, 50 mM NaHCO_3_ and 200 mM NaCl). ChIP-treated DNA was then treated with RNaseA and Proteinase K, purified by phenol‒chloroform extraction and ethanol precipitation, and finally dissolved in TE. To perform ChIP‒qPCR, the amount of purified DNA was quantified by qPCR. The sequences of the primers used are listed in Table S4.

To perform ChIP-seq analysis, the libraries were prepared from 500 pg of immunoprecipitated DNA fragments using a KAPA Hyper Prep Kit (KAPA Biosystems) and subjected to single-end sequencing for 93 cycles on a HiSeq2500 (Illumina). All sequence reads were extracted in FASTQ format using BCL2FASTQ Conversion Software 1.8.4 in the CASAVA 1.8.2 pipeline (Illumina). Mapping was performed by Bowtie2 (version 2.2.6)^47^ using the reference human genome NCBI build 37 (hg19), and ChIP peaks were called by MACS (version 1.4.2)^48^. The data have been deposited in the Gene Expression Omnibus database (accession number GSE227357) and are presented in Table S2.

### Immunofluorescence staining

After treating cells with the reagents under the conditions shown in the figures, the cells were fixed with 4% formaldehyde for 10 min at room temperature, washed three times with PBS, and permeabilized with 0.3% Triton X-100 and 5% goat serum in PBS for 1 h at room temperature. The cells were treated with the antibodies indicated in the figures for 1 h at room temperature. After washing three times with PBS, the cells were incubated with Alexa Fluor 488-, Alexa Fluor 546- or Alexa Fluor 594-conjugated secondary antibodies (Invitrogen) for 1 h at room temperature. The nuclei were stained with 4’,6’-diamidino-2-phenylindole (DAPI) (Dojindo). After washing with PBS three times, the cells were sealed with fluorescence mounting medium (Dako). Fluorescence images were captured with a Zeiss LSM900 confocal microscope, and representative images are shown. To quantify the number, fluorescence intensities and diameters of p62-positive puncta in more than 400 cells, we used the ‘Analyze particle’ function in Fiji (version 2.3.0, ImageJ version 1.53q)^49, 50^. Colocalization analysis was carried out using the ‘Plot Profile’ function in Fiji.

### Plasmids

The 3xFlag-hGABARAPL1 plasmid was generated by subcloning the PCR-amplified human GABARAPL1 cDNA into a p3xFLAG-CMV 10 vector (Sigma). HA-MAP1LC3A, HA-MAP1LC3B, HA-MAP1LC3C, HA-GABARAPL1, HA-GABARAP, HA-GABARAPL2 and Myc-hULK1 were purchased from Addgene (plasmid #137756, #137757, #137758, #137759, #137760, #137761, and #31961, respectively)^51, 52^. Flag-hp62 was kindly provided by Dr. Masaaki Komatsu^53^.

### Generation of the p62 reporter plasmid and its stable expression cell line

The p62 reporter plasmid pmRFP-EGFP-mp62 (RG-p62) was constructed using the Gateway system (Thermo Fisher Scientific). The GFP-mp62/Sqsts1 gene, in which mouse p62/Sqstsm1/A170 cDNA (NM_011018) was conjugated at the C-terminus of the EGFP gene^23^, was amplified from the pEGFP-mp62 vector^54^ using GFP-gwL-F and p62/A170-gwL-R primer sets (Table S2). The GFP-mp62 fragment was further amplified and the attL1/L2 sequence was conjugated using an attL1-F and attL2-R primer set (Table S4) as described previously^23^. The amplified attL1-GFP-mp62-attL2 fragment was cloned into a pmRFP-N1-DEST vector^24^ using the Gateway system. The pRFP-EGFP-mp62 plasmid was stably transfected into Neuro2a cells. RFP- and GFP-positive single clones were picked after visualization with fluorescence microscopy and subcloned at least twice.

### p62-dependent aggrephagy assay

Neuro2a cells stably expressing RG-p62 were treated as described in the figure legends, fixed with 4% formaldehyde for 10 min at room temperature, and washed three times with PBS. The nuclei were stained with 4’,6’-diamidino-2-phenylindole (DAPI) (Dojindo). After washing with PBS three times, the cells were sealed with fluorescence mounting medium (Dako). Fluorescence images were captured with a Zeiss LSM900 confocal microscope, and representative images are shown. The fluorescence intensities of RFP and GFP were quantified in at least four fields of view under all experimental conditions (total cell numbers > 38 cells per condition) using Fiji, and the ratios of GFP/RFP are presented.

### Coimmunoprecipitation experiments

293T cells were transfected with the indicated plasmids. One day after transfection, the cells were treated with 1 µM MG132 for 16 h, and whole-cell extracts were prepared in lysis buffer (20 mM Tris–HCl [pH 8.0], 100 mM NaCl, 1 mM EDTA, 0.1% NP-40, 1 mM NaF, 1 mM Na_3_VO_4_, 10 mM β-glycerophosphate, 10 µM MG132, and protease inhibitor cocktail). Proteins were immunoprecipitated using an anti-HA antibody (3F10; Sigma) and Dynabeads protein G. Immunoprecipitated proteins were washed 3 times with lysis buffer. The immunoprecipitated samples were denatured with SDS sample buffer and subjected to immunoblot analysis.

### Statistical analysis

The statistical significance of repeated measurements was evaluated using ANOVA–Tukey and Welch t test. These analyses were performed using Microsoft Office Excel (Microsoft) and GraphPad Prism 9 (GraphPad Software, version9.5.1). All the values are presented as the mean ± standard deviation (SD) on the basis of at least three independent experiments.

### Supplementary Information

Supplementary Figures.

Supplementary Table S1.

Supplementary Table S2.

Supplementary Table S3.

Supplementary Table S4.

## Data Availability

Data for the DNA microarray (GSE227232) and ChIP-sequence analysis (GSE227357) are available in the Gene Expression Omnibus database. Other data will be made available on request to AK.

## Abbreviations

ARE: Antioxidant response element
ERAD: Endoplasmic reticulum-associated protein degradation
GABARAPL1: GABA(A) receptor-associated protein-like 1
NRF1: NFE2-related factor 1
NFE2L1: NFE2-like 1
TSS: Transcriptional start site
ULK1: Unc-51-like autophagy activating kinase 1
UPS: Ubiquitin‒proteasome system
