# Supplementary material for: The transcription factor NRF1 (NFE2L1) activates aggrephagy by inducing p62 and GABARAPL1 after proteasome inhibition to maintain proteostasis
Source: Sci Rep. 2023 Sep 1;13:14405. doi: 10.1038/s41598-023-41492-9 (PMC10474156; doi:10.1038/s41598-023-41492-9)

Figure S1

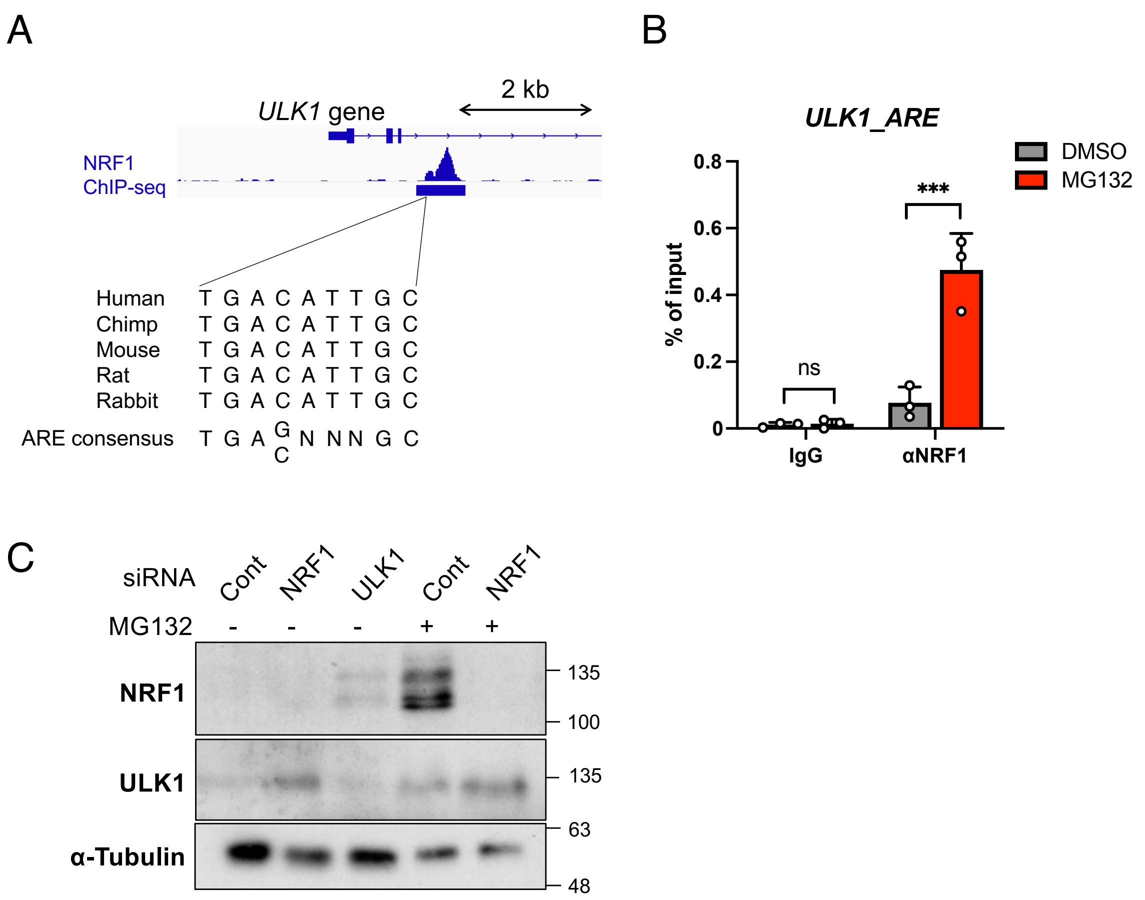


**Supplementary figure 1. After proteasome inhibition, NRF1 directly upregulates ULK1 expression at the mRNA level but not at the protein level.** (A and B) The recruitment of NRF1 to the promoter region of the ULK1 gene in HCT116 cells after proteasome activity was impaired. As shown in (A), ChIP-seq signals of NRF1 are shown on the genome loci of ULK1 in the human genome using Integrative Genomics Viewer (IGV). As shown in (B), HCT116 cells were treated with MG132 (1 μM) for 16 h and then subjected to ChIP–qPCR analysis. (C) NRF1 knockdown did not alter the protein levels of ULK1 in HCT116 cells treated with MG132 (1 μM) for 16 h. (B) ANOVA followed by Tukey's test, mean ± SD, ***: p<0.005, ns: not significant (n=3).

# Figure S2

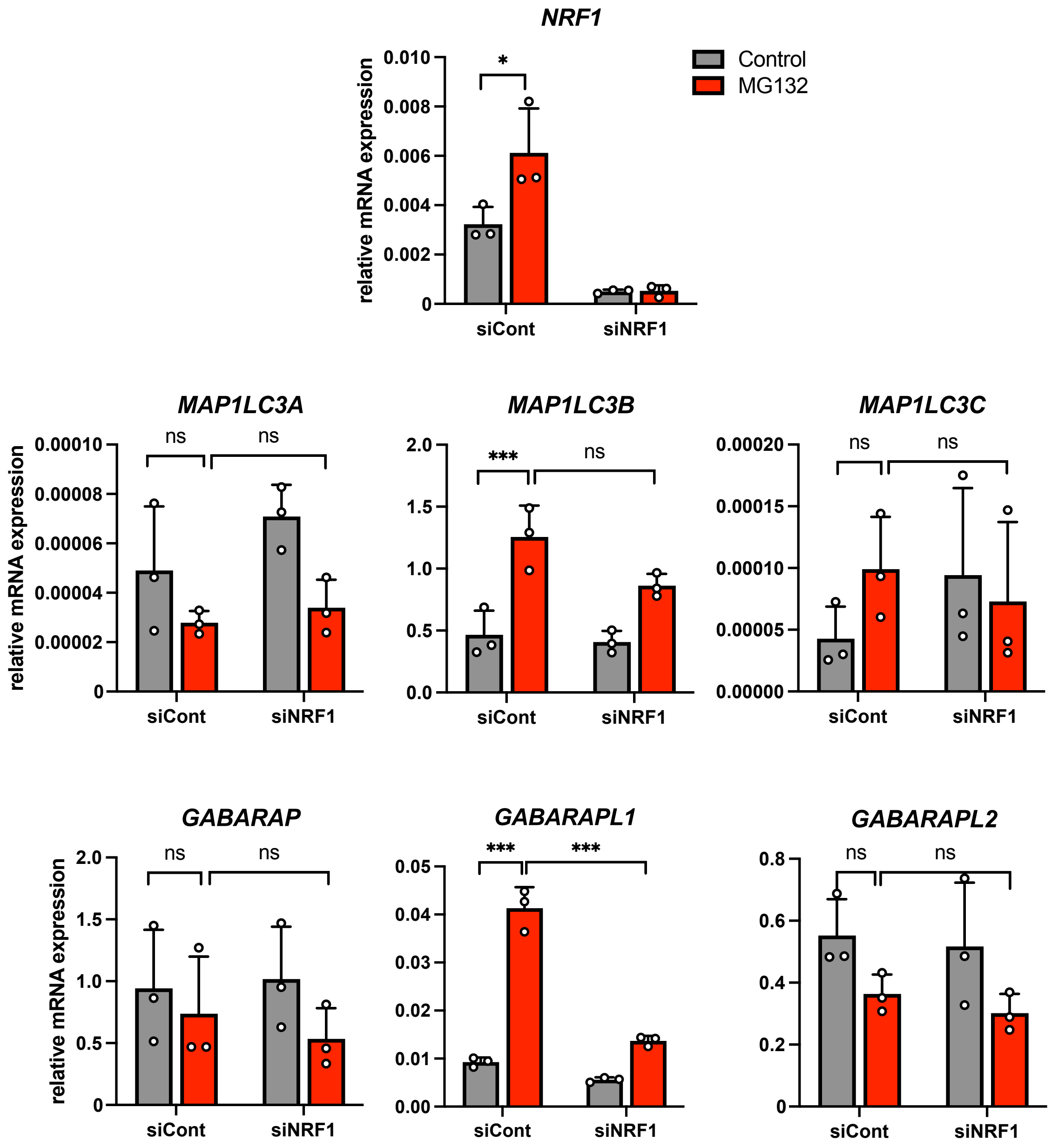


**Supplementary figure 2. NRF1 selectively upregulates the gene expression of GABARAPL1, but not other ATG8 family genes, in response to proteasome inhibition.** siRNA-mediated NRF1 knockdown and RT–qPCR analysis with HCT116 cells were performed, as described in the legend of Figure 3A. ANOVA followed by Tukey's test: mean ± SD, *: $p<0.05$, ***: $p<0.005$ (n=3).

# Figure S3

Fig1A

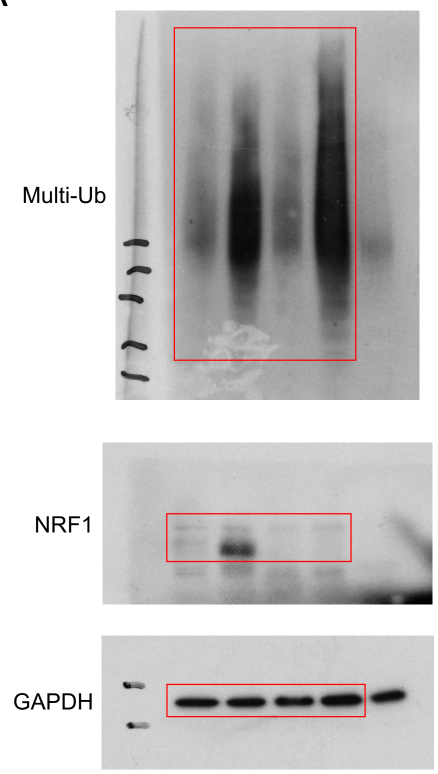


Fig1D

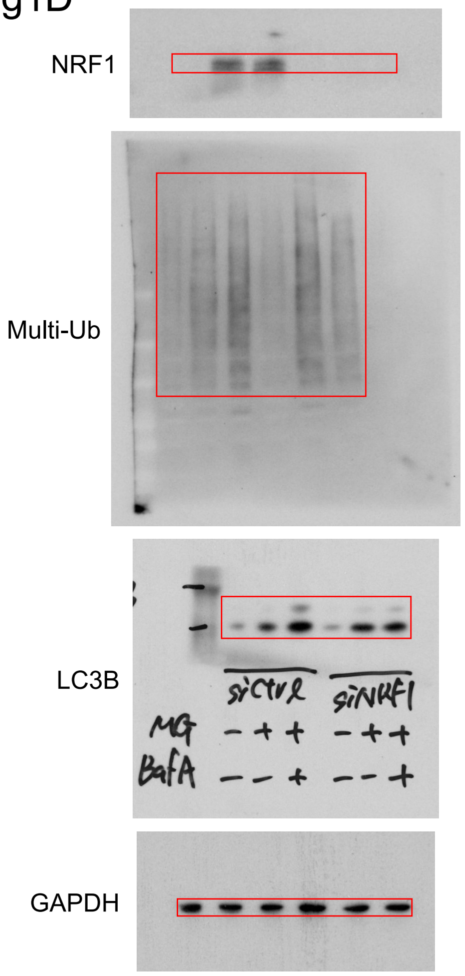


Fig4A

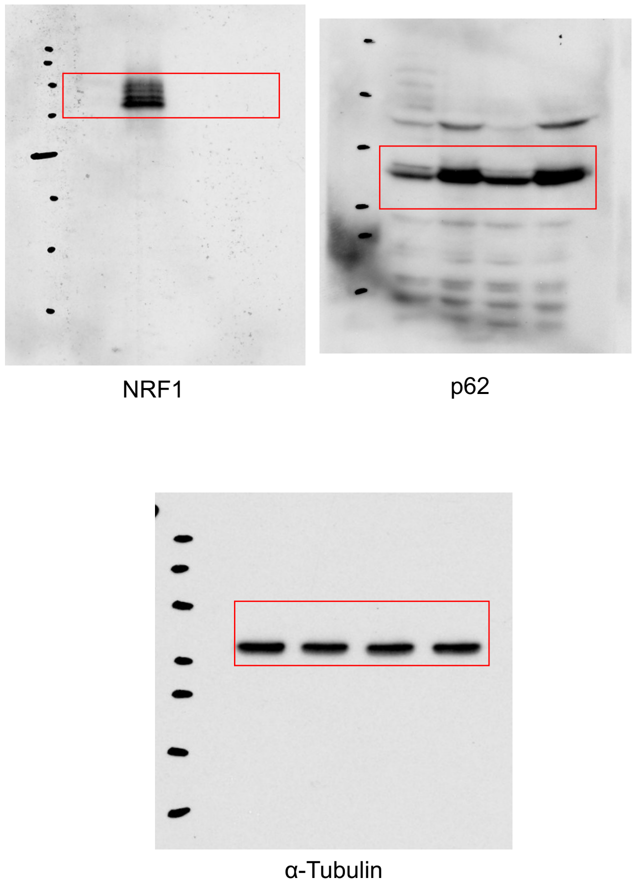


Fig4C

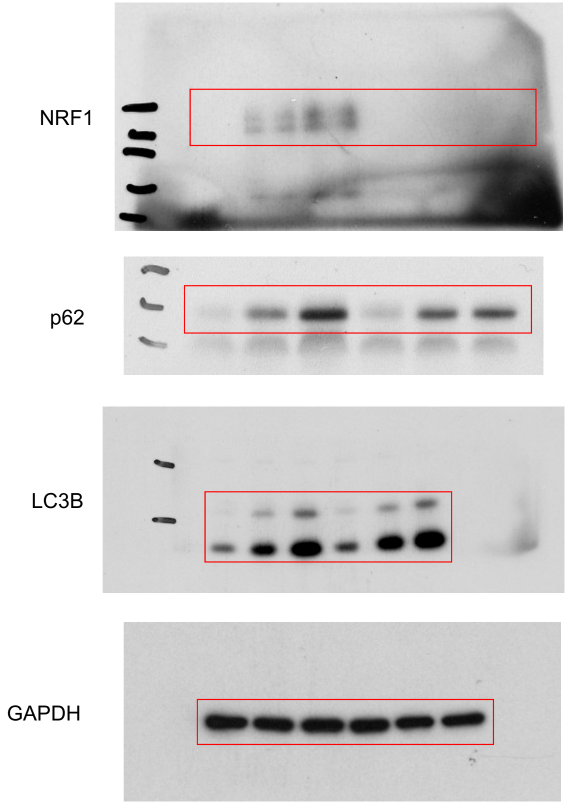


**Supplementary figure 3. Uncropped immuno-blots.**

# Figure S3 continued

Fig4D

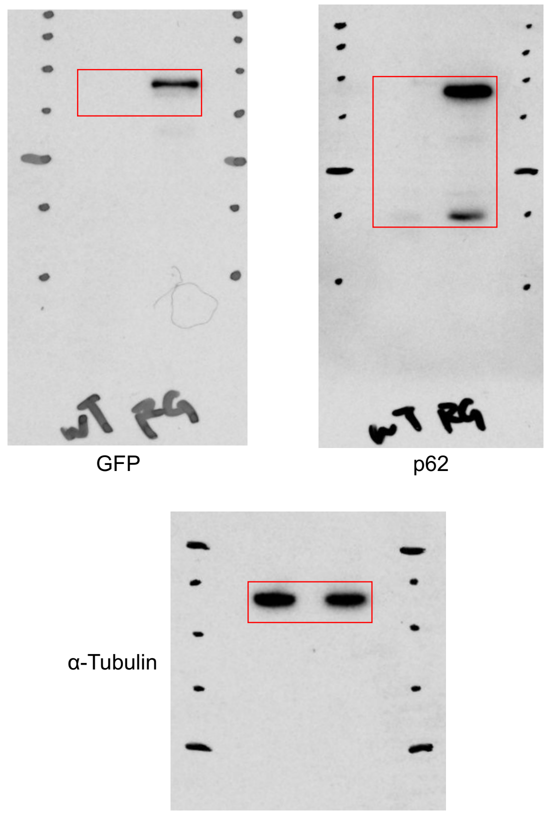


Fig4E

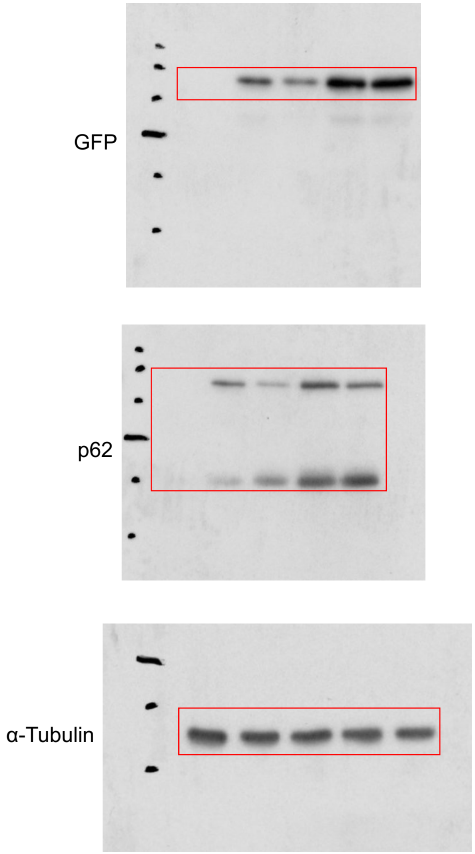


Fig5E

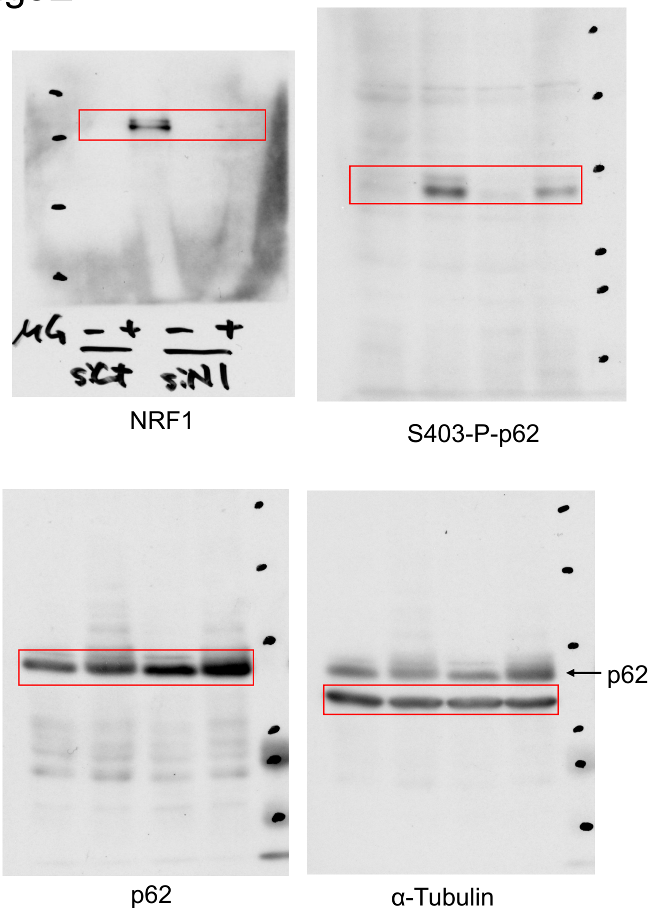


Fig6A

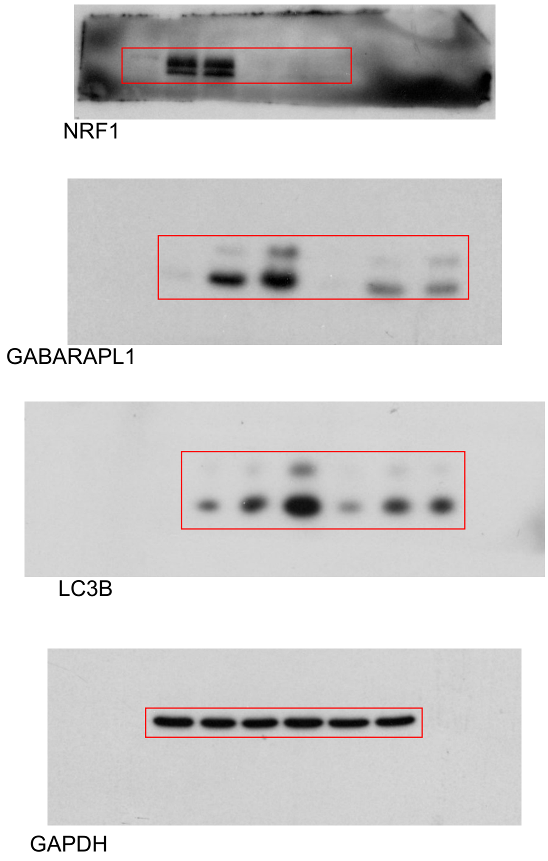

# Figure S3 continued

## Fig6C

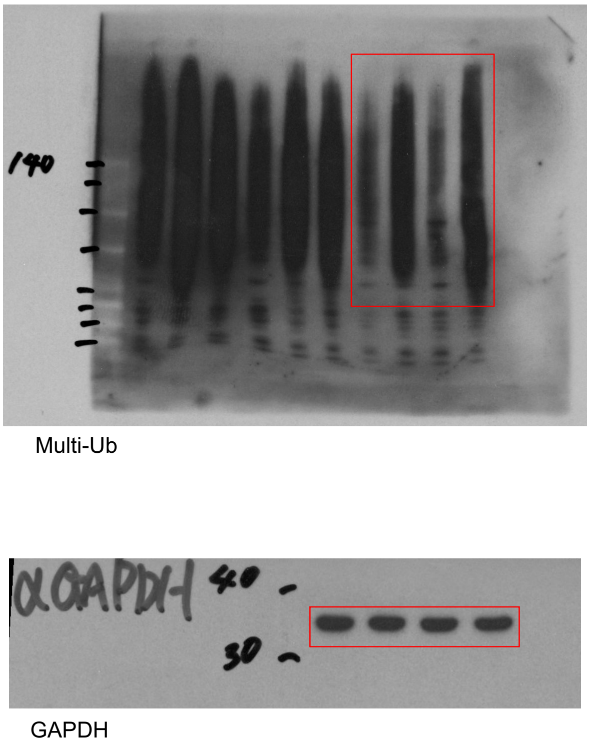


## Fig6D

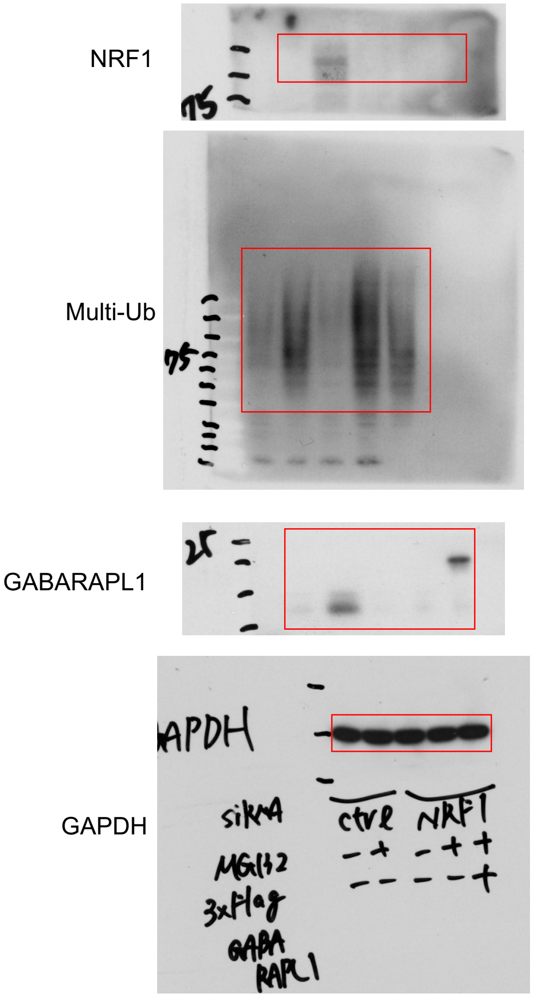


## Fig6E

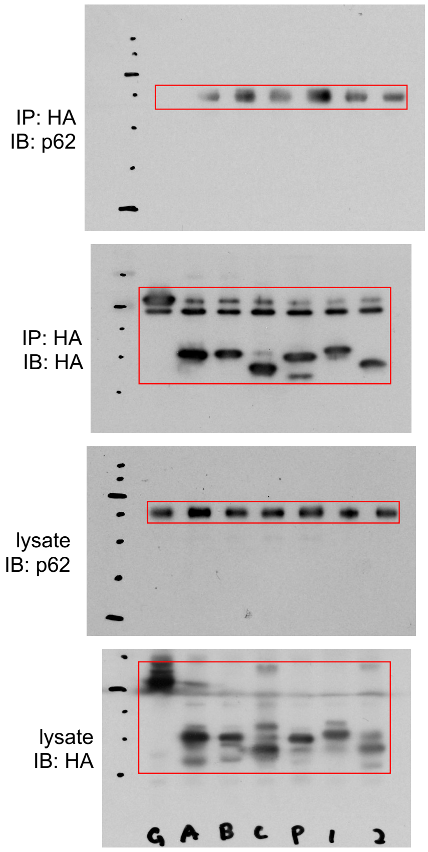


## Fig6G

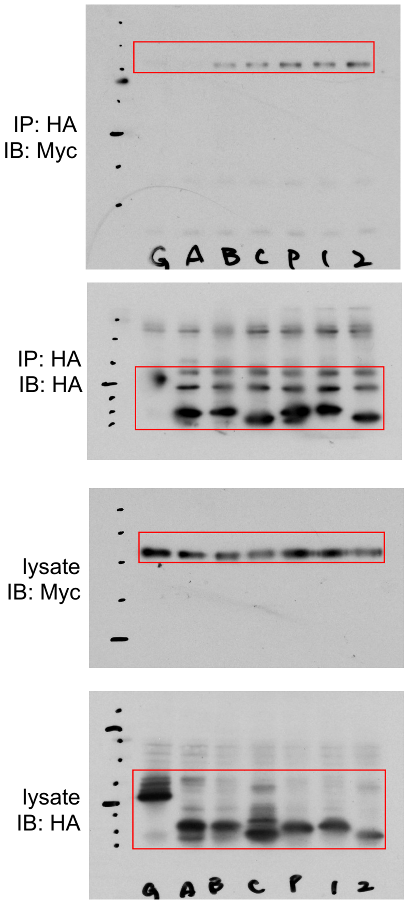

# Figure S3 continued

FigS1C

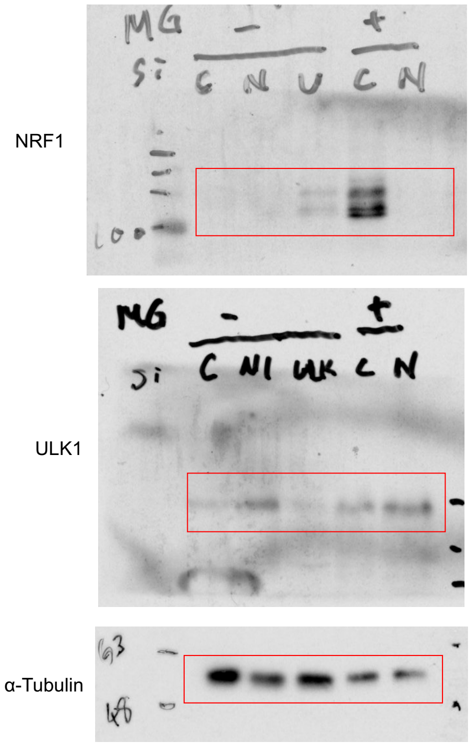

Supplement: Supplementary file 1 — Supplementary Figures. [file 41598_2023_41492_MOESM1_ESM.pdf]
